# Clinical course for pancreatic necrosis and pancreatic pseudocysts due to severe acute or chronic pancreatitis

**DOI:** 10.1177/17562848241301945

**Published:** 2024-11-23

**Authors:** Stefano Fusco, Greta M. Hanke, Karsten Büringer, Lisa Minn, Gunnar Blumenstock, Ulrike Schempf, Martin Götz, Nisar P. Malek, Dörte Wichmann, Christoph R. Werner

**Affiliations:** Section of Gastroenterology, Gastrointestinal Oncology, Hepatology, Infectiology and Geriatrics, Department of Internal Medicine I, University Hospital of Tübingen, Otfried-Müller-Str. 10, Tübingen 72076, Germany; Department of Nephrology, Medical Faculty, Heinrich-Heine University, Düsseldorf, Germany; Section of Gastroenterology, Gastrointestinal Oncology, Hepatology, Infectiology and Geriatrics, Department of Internal Medicine I, University Hospital of Tübingen, Tübingen, Germany; Section of Gastroenterology, Gastrointestinal Oncology, Hepatology, Infectiology and Geriatrics, Department of Internal Medicine I, University Hospital of Tübingen, Tübingen, Germany; Department of Clinical Epidemiology, Eberhard-Karls-University, Tübingen, Germany; Section of Gastroenterology, Gastrointestinal Oncology, Hepatology, Infectiology and Geriatrics, Department of Internal Medicine I, University Hospital of Tübingen, Tübingen, Germany; Division of Gastroenterology, Klinikum Böblingen, Böblingen, Germany; Section of Gastroenterology, Gastrointestinal Oncology, Hepatology, Infectiology and Geriatrics, Department of Internal Medicine I, University Hospital of Tübingen, Tübingen, Germany; Section of Gastroenterology, Gastrointestinal Oncology, Hepatology, Infectiology and Geriatrics, Department of Internal Medicine I, University Hospital of Tübingen, Tübingen, Germany; Section of Gastroenterology, Gastrointestinal Oncology, Hepatology, Infectiology and Geriatrics, Department of Internal Medicine I, University Hospital of Tübingen, Tübingen, Germany

**Keywords:** endoscopy, LAMS, necrosectomy, pancreatitis, pseudocyst, walled-off necrosis

## Abstract

**Background:**

The acute and chronic pancreatitis (CP) can lead to severe complications like walled-off necrosis, large symptomatic pseudocyst or multiorgan failure. The treatment of these complications is multivariate and can differ from conservative, symptomatic treatment or minimal-invasive, endoscopic transgastral stenting to transgastral necrosectomy.

**Objectives:**

This study aims to analyse the clinical course for patients that develop local complications of severe pancreatitis.

**Design:**

This is a retrospective observational single-centre study on 46 patients with severe pancreatitis.

**Methods:**

In this retrospective single-centre study, 46 out of 474 inpatients from January 2014 to December 2020, who were treated because of an acute or CP, developed acute pancreatitis complications and could be included. We analysed and compared the clinical course of different treatments (lumen apposing metal stents, transgastral double pigtail stent, endoscopic retrograde cholangiopancreatography, operation, conservative treatment) and different complications (walled-off necrosis (WON), pancreatic pseudocyst (PPC)).

**Results:**

Forty-six patients developed an acute complication due to severe pancreatitis. Twenty-seven patients developed a WON, while 19 patients suffered from PPC. 48% of the whole cohort had an alcoholic aetiology of pancreatitis. 78% were treated with antibiotics, 48% suffered from infected pancreatitis and 22% needed intensive care treatment. WON patients more often had a longer hospitalization of more than 21 days. PPC patients were correlated with an alcoholic aetiology, whereas WON patients were inversely correlated with an alcoholic aetiology. Increased lactate dehydrogenase, lipase, and C-reactive protein levels as well as leucocyte count could be associated with a higher probability to exhibit a WON instead of another local complication. The mortality rate was low with 7% in our study.

**Conclusion:**

WON and PPC differ in certain patients and laboratory characteristics such as aetiology, elevated laboratory values, antibiotic treatment or the duration of hospitalization. Invasive treatment is not required in all severe pancreatitis cases.

## Introduction

Pancreatitis is a potentially serious inflammatory disease that can lead to multiple organ failure. It is divided into acute and chronic forms. Acute pancreatitis (AP) can be classified into three distinct categories, namely mild, moderate and severe forms. The chronic pancreatitis (CP)^1,2^ is a pathologic fibro-inflammatory syndrome of the pancreas that develops persistent pathologic responses to parenchymal injury or stress. Common features of established and advanced CP include pancreatic atrophy, fibrosis, pain syndromes, duct distortion and strictures, calcifications, pancreatic exocrine dysfunction, pancreatic endocrine dysfunction and dysplasia.^3^ AP is one of the most common gastrointestinal disorders requiring acute hospitalization worldwide,^4–7^ with a reported increasing annual incidence of 13–45 cases per 100,000 persons, whereas CP ranges from 5 to 12 cases per 100,000 persons.^8^ Local complications are peripancreatic fluid collections, pancreatic and peripancreatic necrosis (sometimes infected), and formation of pseudocysts and walled-off necrosis (WON).^2^

AP is diagnosed when at least two of the following three criteria are met: (i) upper abdominal pain (typically radiating belt-like to both sides, excluding pain from other conditions; (ii) an increased level of serum lipase exceeding three-fold the upper limit of normal; (iii) typical findings in sectional image studies, such as transabdominal sonography, computed tomography (CT) or magnetic resonance imaging (MRI) suggestive of AP.^2,9–11^ Definitive CP can be diagnosed by imaging criteria (CT, MRI, endoscopic ultrasound (EUS)) alone, whereas probable CP requires both clinical features and imaging criteria. Cross-sectional imaging should be used first; EUS is needed only when CT or MRI are inconclusive or to perform therapeutic interventions.^12^ The diagnosis of CP is based on pancreatic calcifications, ductal dilatation or distortion and glandular atrophy visualized by imaging with CT, MRI or EUS or by biopsy demonstrating widespread fibrosis.^13,14^ One of the ways in which CP can be manifested is through the formation of pancreatic duct stones. Pancreatic duct stones are frequently present without any acute exacerbations. However, it is not clearly elucidated whether pancreatic duct stones can lead to an acute exacerbation of the CP with abdominal pain, nausea and vomiting and sometimes increased lipase levels in the blood.^15,16^ Another typical but rare complication of CP is a common bile duct stricture (CBDS) due to pancreatic parenchyma atrophy.^17,18^

Mild AP has no major local or systemic complications, whereas in around 20% of all patients, a severe clinical course develops, associated with systemic inflammation even leading to multi-organ failure and significant local damage.^19^ Pancreatic fluid collection (PFC) and WON are frequent complications of severe AP. Symptomatic PFC requires drainage; options include surgical, percutaneous or endoscopic approaches. With innovative endoscopic tools, minimally invasive endoscopic drainage has become the preferable approach.

Endoscopic transmural puncture for necrotic pancreatitis with stent placement may provide access for drainage and decompression. In cases of more complex collections, transluminal instrumentation with lavage, debridement and necrosectomy may be required.^20^ In patients with sterile or infected pancreatic necrosis and persistent unwellness marked by abdominal pain, nausea, vomiting and nutritional failure, or with associated complications, including gastrointestinal luminal obstruction, biliary obstruction, recurrent AP, fistulas, or persistent systemic inflammatory response syndrome, endoscopic drainage and/or debridement may be required.^21^ An EUS-guided approach utilizing the Seldinger technique is the preferred method for endoscopic intervention. Lumen-apposing metal stents (LAMS) have been demonstrated to be both efficacious and safe and can be employed particularly in the treatment of large infected pseudocysts and WON.^22^ Angadi et al.^23^ indicated in a prospective randomized trial that the laparoscopic drainage of a WON was not superior to EUS-guided transmural drainage with placement of multiple pigtail stents (PS) or LAMS. A recently published Spanish prospective, multicentre, open-label trial did not demonstrate superiority for LAMS over double pigtail stent (DPS) for WON therapy.^24^ The two techniques, LAMS and DPS, can be considered equally effective, with each technique offering distinct advantages. LAMS is more favourable in certain cases due to its larger diameter, which allows for direct endoscopic necrosectomy. By contrast, DPS can be applied less invasively and is less expensive.

The advancement in endoscopic techniques has led to the replacement of surgery as the primary treatment for WON with endoscopic procedures.^24,25^

## Methods

### Study setting and patient selection

Primarily, all patients suffering from acute or CP from the database of the University Hospital of Tübingen, a tertiary centre in south western Germany, were included.

The study was approved by the local Institutional Review Board, the Ethics Committee of the Medical Faculty of the University of Tübingen (IRB number: 279/2020BO2) on 5 May 2020 and was conducted in accordance with the current version of the declaration of Helsinki.

All patients treated at our University Hospital between January 2014 and December 2020 were considered for inclusion in this retrospective study, provided that the following criteria were met: the patient was aged 18 years or older, the patient received inpatient care, the patient’s vital parameters and pain score were documented, the patient’s blood values (e.g. haemoglobin, C-reactive protein (CRP), leucocytes) were documented and the patient was experiencing an acute or acute-on-chronic episode of pancreatitis.

Of the 474 patients, 100 were admitted as inpatients due to pancreatitis, either as a result of a general practitioner referral or via the emergency department. A total of 60 patients were included in the study, as 40 of the 100 patients had no clinically relevant complications as defined by the study criteria. To be included in the study, patients had to have been hospitalized due to a pancreatitis episode (either acute or chronic) and have a confirmed local complication of the pancreatitis. A total of 27 patients (45%) had necrotizing pancreatitis, 19 patients (32%) had a pseudocyst, 9 patients (15%) had common bile duct stenosis (CBD stenosis) caused by pancreatitis and 5 patients (8%) had pancreatic duct stones due to previous pancreatitis.

**Figure 1.**
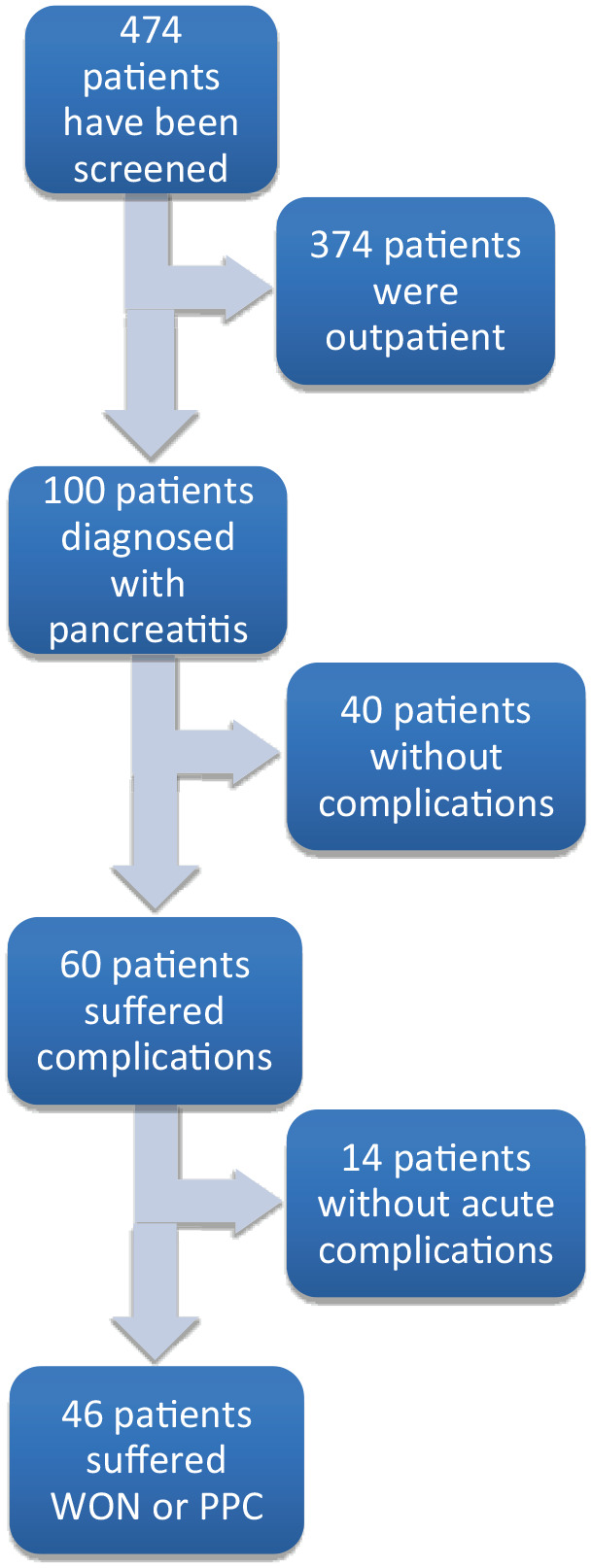
Flowchart of patient identification and inclusion.

The parameters to be analysed were recorded using the patients’ electronic inpatient medical records. The parameters were collected utilizing the hospital’s internal SAP (SAP Deutschland SE & Co. KG, Walldorf, Germany) and Lauris (Nexus AG, Donaueschingen, Germany) operating systems. All parameters and findings were initially documented in an Excel spreadsheet (Microsoft, Portland, OR, USA) and subsequently transferred to an SPSS spreadsheet (IBM Corp., Armonk, NY, USA) for statistical analysis. The flowchart of patient identification and inclusion is presented in Figure 1. A total of 474 patients were screened, of whom 100 patients with elevated lipase were identified as having a confirmed diagnosis of pancreatitis. Of these, 40 patients exhibited no complications, while 60 patients experienced local complications. After excluding patients with chronic complications (*n* = 14), 46 patients with acute complication were included in the study.

### Statistical analysis

The statistical analysis was conducted using the Statistical Software Package for Social Sciences (SPSS) version 29.0 for Windows (IBM Corp.). For baseline descriptive statistics, mean with standard deviation was employed for variables exhibiting a parametric distribution, whereas median with 25th and 75th percentiles was utilized for those displaying a non-parametric distribution. Outcome variables were reported as medians with adjusted 95% confidence intervals. The Mann–Whitney *U* test, the χ^2^-test and the Fisher exact test were used to compare groups where appropriate. The significance level was defined as *p* ⩽ 0.05. The primary endpoints of this single-centre, retrospective study were the analysis of the clinical course of severe pancreatitis, as well as the identification of potential risk factors and predictors of WON and pancreatic pseudocyst (PPC).

The reporting of this study conforms to the Strengthening the Reporting of Observational Studies in Epidemiology (STROBE) statement.^26^ We confirm that the results are reproducible with the help of the specified methods and patients. The patients were collected consecutively from the electronic patient records after querying the operation and procedure codes via internal controlling. The sample size was not calculated.

## Results

### Study population characteristics and demographics

**Figure 2.**
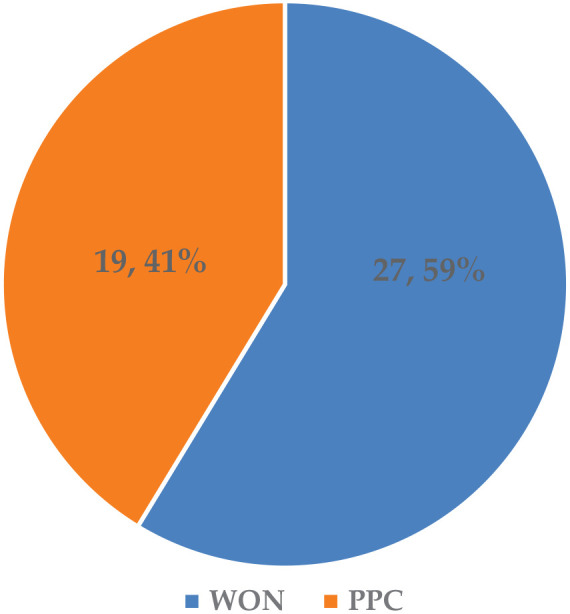
Frequency of complications of chronic or acute pancreatitis (*n*; %).

A total of 46 adult patients presenting with either an elevated lipase level or abdominal pain and a suspected diagnosis of pancreatitis were included in this study. The present study was limited to patients with a diagnosed WON or PPC, as these are the most common acute complications of pancreatitis (see Figure 2). Of the 46 patients included in the study, 27 (59%) exhibited WON, while 19 (41%) developed PPCs.

Table 1 presents the characteristics and demographics of the patient cohort. The male gender was overrepresented numerically, although the small sample size precludes a definitive conclusion (*p* = 0.84). The median age differs significantly between the PPC subcohort (50 years) and the remainder of the patient cohort (61 years; *p* = 0.019). No significant difference was observed in body mass index (BMI) between the groups, although the WON and pancreatic duct lithiasis (PDS) cohorts exhibited a mean BMI exceeding 25 kg/m^2^, indicative of overweight, while the PPC and CBD stricture patients demonstrated a mean BMI within the normal range.

**Table 1.** Patients’ characteristics.

Variables	WON	PPC	Total	*p* Values
Cohort size, *n*	27	19	46	
Patients’ characteristics				
Sex, *n* (%)				
Female	8 (30)	7 (37)	15 (33)	0.617
Male	19 (70)	12 (63)	31 (67)	0.617
Age, median, IQR	60 (50–68)	50 (42–56)	55 (48–67)	0.159
BMI, mean ± SD	26.0 ± 5.7	23.4 ± 6.5	24.5 ± 5.7	0.098
The period from the onset of pancreatitis to intervention (d), mean ± SD	38.9 ± 9.9	46.3 ± 9.5	42.0 ± 10.3	0.014
Laboratory values (median, IQR)				
Lipase (U/L)	383 (82–2009)	80 (60–212)	124 (29–952)	0.014
CRP (mg/dL)	24.3 (13–32)	12.0 (2–18)	16.0 (2.2–24.3)	<0.001
WBC (Gpt/L)	20.6 (14.6–26.1)	11.9 (6.8–14.2)	12.7 (9.5–20.5)	<0.001
LDH (U/L)	384 (212–488)	216 (148–272)	247 (180–365)	0.002
Comorbidities, *n* (%)				
Cardiac	17 (63)	11 (58)	28 (61)	0.736
Pulmonary	8 (30)	3 (16)	11 (24)	0.289
Haematologic	6 (22)	1 (50)	7 (15)	0.120
Gastrointestinal	17 (63)	13 (68)	30 (65)	0.710
Malignancy	5 (19)	4 (21)	9 (20)	0.836
Alcohol abuse, *n* (%)				
Yes	10 (37)	14 (74)	24 (52)	0.014
No	17 (63)	5 (26)	22 (48)	0.014
Nicotine consumption, *n* (%)				
Yes	7 (26)	11 (58)	18 (39)	0.029
No	20 (74)	8 (42)	28 (61)	0.029

BMI, body mass index; CRP, C-reactive protein; IQR, interquartile range; LDH, lactate dehydrogenase; PPC, pancreatic pseudocyst; SD, standard deviation; WON, walled-off necrosis.

The most common comorbidities are cardiac and gastrointestinal diagnoses. It is noteworthy that there was a significant discrepancy in the prevalence of alcohol abuse between the WON and PPC cohorts (*p* = 0.015). While a greater proportion of WON patients demonstrated abstinence from alcohol (17/27; 63%), the majority of PPC patients (14/19; 74%) exhibited a positive history of alcohol consumption. Similarly, a correlation can be observed with nicotine abuse. Of the WON cohort, 20/27 (74%) had never smoked, while 11/19 (58%) of the PPC cohort were current or former smokers (*p* = 0.03).

The predominant site of fluid collections or necrotic tissue was intrapancreatic (33/46; 72%). In approximately two-thirds of cases (17/27; 63%) within the WON cohort, lesions were observed both intra- and extrapancreatic concurrently. By contrast, the PPC cohort predominantly exhibited an intrapancreatic localization.

With regard to the laboratory values, a higher median lipase level was observed at the time of hospital admission in the WON group, with a value of approximately 380 U/L. The WON and PDS groups exhibited the highest maximum lipase levels, at 3429 and 4101 U/L, respectively. The WON group exhibited a higher median value of CRP, at 24 mg/dL. The remaining three cohorts exhibited CRP median values between 2 and 12 mg/dL. A significant and relevant leucocytosis was observed more frequently in the WON group (20.6 Gpt/L) than in the other three groups (median ranging from 9.4 to 11.9 Gpt/L; *p* < 0.001). Additionally, a relevantly elevated LDH concentration was only exhibited in the WON group (median 384 vs 216; *p* = 0.002).

The interval between the onset of pancreatitis and intervention (in the invasive-treated patients) differed significantly between the two cohorts (WON vs PPC). While the WON cohort exhibited a mean of approximately 39 days between the onset of symptoms and intervention, the PPC cohort demonstrated a period of 46 days between the onset of symptoms and intervention.

### Aetiology of pancreatitis

The study population exhibited a range of pancreatitis aetiologies, as detailed in Table 2. Of the 60 cases, 28 (47%) were classified as ethyltoxic, 8 (13%) as biliary, 3 (5%) as autoimmune and 3 (5%) as iatrogenic (post-endoscopic retrograde cholangiopancreatography (ERCP)). Furthermore, one subject (2%) was found to have an anatomical variant (pancreas divisum), which was identified as the most probable cause of pancreatitis. In 17/60 (28%) of patients, no definitive cause for pancreatitis could be identified. It is noteworthy that almost 74% (14/19) of the PPC cohort exhibited an ethyl toxic aetiology, whereas less than 30% (8/27) of the WON patients had an ethyl toxic cause. An idiopathic aetiology was observed in every subgroup, with a total rate of approximately 28%.

**Table 2.** Peri-procedural characteristics.

Variables	WON	PPC	Total	*p* Values
Cohort size, *n*	27	19	46	
Pancreatitis: aetiology, chronicity, localization of lesion				
Pancreatitis aetiology, *n* (%)				
Biliary	6 (22)	1 (5)	7 (15)	0.120
Ethyltoxic	8 (30)	14 (74)	22 (48)	**0.003**
Iatrogenic	3 (11)	0 (0)	3 (7)	0.562
Autoimmunological	0 (0)	0 (0)	0 (0)	
Anatomical	1 (4)	0 (0)	1 (2)	0.763
Unknown	9 (33)	4 (21)	13 (28)	0.374
Chronicity, *n* (%)				
Acute	16 (59)	5 (26)	21 (46)	**0.027**
Chronic	11 (41)	14 (74)	25 (54)	**0.027**
Localization of fluid/necrosis				
Intrapancreatic	9 (33)	18 (95)	27 (59)	**0.030**
Peripancreatic	1 (4)	0 (0)	1 (2)	**0.030**
Both	17 (63)	1 (5)	18 (39)	**0.045**
Treatment characteristics				
Antibiotic treatment, *n* (%)				
Yes	24 (89)	12 (63)	36 (78)	**0.038**
No	3 (11)	7 (37)	10 (22)	**0.038**
1 antibiotic agent	6 (25)	10 (83)	16 (44)	**<0.001**
⩾2 antibiotic agents	18 (75)	2 (17)	20 (56)	**<0.001**
Carbapenems^a^	18 (75)	1 (8)	19 (53)	**<0.001**
Cephalosporins^a^	8 (33)	3 (25)	11 (31)	0.289
Penicillins^a^	3 (13)	1 (8)	4 (11)	0.499
Fluorchinolones^a^	7 (29)	6 (50)	13 (36)	0.683
Other^a^	16 (67)	3 (25)	19 (53)	0.107
Duration of antibiosis				
⩽7 days, *n* (%)	1 (4)	5 (42)	6 (17)	**0.001**
>7 days, *n* (%)	23 (96)	7 (58)	30 (83)	**0.001**
ICU, *n* (%)	9 (33)	1 (5)	10 (22)	**0.006**
Thrombosis, *n* (%)	8 (30)	1 (5)	9 (25)	**0.004**
Infected pancreatitis, *n* (%)	18 (67)	10 (53)	28 (61)	**0.004**
Inpatient stay, d (median, IQR)	25 (3–137)	5 (2–19)	11 (2–137)	
⩽21 days, *n* (%)	13 (48)	19 (100)	32 (70)	**<0.001**
>21 days, *n* (%)	14 (52)	0 (0)	14 (30)	**<0.001**
Endoscopic intervention (%)^b^				
No intervention	10 (37)	2 (11)	12 (26)	**0.005**
Intervention	17 (63)	17 (89)	34 (74)	**0.005**
EUS	7 (41)	11 (65)	18 (39)	0.138
ERC	10 (59)	6 (35)	16 (35)	0.095
LAMS (HotAxios)	2 (12)	5 (29)	7 (15)	**0.045**
Double pigtail	3 (18)	5 (29)	8 (17)	0.112
CBD SEMS	0 (0)	0 (0)	0 (0)	
CBD plastic stent	2 (12)	6 (35)	8 (17)	**0.021**
Other	10 (59)	1 (6)	11 (24)	0.067
Outcome				
Death, *n* (%)	2 (7)	1 (5)	3 (7)	0.835

a Can exceed the total number due to combinations of antibiotic treatment.

b Can exceed the total number due to combinations of interventions.

The bold entries indicate a statistical significance (*p* <0.05).

CBD, common bile duct; ERC, endoscopic retrograde cholangiography; EUS, endoscopic ultrasound; ICU, intensive care unit; IQR, interquartile range; LAMS, lumen apposing metal stent; PPC, pancreatic pseudocyst; SEMS, self-expanding metal stent; WON, walled-off necrosis.

Neither cohort of patients displayed evidence of an autoimmune aetiology of pancreatitis.

A total of 46% (21/46) of the complications were associated with AP, while 54% (25/46) were attributed to CP.

### Treatment characteristics

For details regarding the treatment administered, please refer to Table 2. Of the patients included in the study, 78% (36/46) were treated with antibiotics. The majority of these patients were part of the WON cohort, with an 89% treatment rate (24/27), compared to a 63% treatment rate (12/19) in the PPC cohort. A notable discrepancy was observed between the WON and PPC groups with respect to the number of antibiotic drugs utilized. The WON cohort exhibited a higher prevalence of treatment with at least two antibiotic agents (75%, 18/24), compared to a mere 17% in the PPC cohort (2/19).

A total of 75% (18/24) of WON patients were prescribed carbapenems, while 50% (6/12) of the PPC cohort received fluorchinolones. Approximately half of the patients received other antibiotics (19/36). The duration of antibiotic therapy was analysed with a cut-off of 7 days. The majority of patients in the WON cohort (23/24; 96%) received antibiotic treatment for a duration exceeding 7 days. Similarly, the patients in the PPC cohort were also predominantly treated for a period of more than 7 days (PPC: 7/12 (58%)).

In 34 out of 46 patients (74%), an endoscopic treatment was initiated. The intervention modalities differed between the four subgroups. The majority of HotAxios stents (5/7) and plastic DPSs (5/8) were applied in patients with peripancreatic cysts. The procedures classified under the heading of ‘other’ included four patients undergoing percutaneous endoscopic necrosectomies, four patients with an inserted duodenal stent, one patient with the treatment of a Whipple procedure and one case of surgical necrosectomy. All five patients with a double pigtail application or a HotAxios stent implantation underwent an endoscopic necrosectomy. If an ERCP with or without papillotomy was necessary in patients with cholestasis due to PPC or WON, a stenting of the CBD had only the aim of treating the cholestasis and not draining the WON or PPC.

The majority of WON patients (59%, 16/27) presented with an acute episode of pancreatitis, whereas the majority of PPC patients (74%, 14/19) exhibited CP. A local thrombosis of visceral veins was diagnosed in nearly 30% (8/27) of the WON cohort, whereas only one patient (5%) of the PPC cohort had a diagnosed thrombosis. The thrombosis occurred either in a mesenteric vein or in a deep venous thrombosis of the lower extremity.

The incidence of infected fluid collections or infected necrosis was comparable between the WON (66%) and PPC (52%) groups. The microbiological sample obtained via EUS fine-needle aspiration revealed the presence of bacteria, including *Escherichia coli, Enterobacter cloacae* and *Enterococcus faecalis*. No significant differences were observed in the bacterial species between the subgroups.

The transfer to an intensive care unit (ICU) was more frequently observed in the WON subgroup of patients, with a rate of 33% (9/27), in comparison to one patient in the PPC cohort (5%). With regard to the administration of vasopressors or invasive ventilation, no notable difference in outcome was observed between ICU patients and those receiving care outside the ICU. The mortality of the three patients was not associated with their ICU stay or any other parameter, respectively, disease characteristic. One patient died as a result of intraperitoneal bleeding and had undergone a necrosectomy 2 weeks prior to his demise. The necrosectomy was performed at an early stage of AP. One patient died of multiorgan failure following the consented deescalation of therapy, which did not involve invasive intervention as this was not requested by the patient. The third patient died from uncontrollable septic shock in a late stage of an infected PPC that was diagnosed via fine needle aspiration. Two of the deceased patients were included in the WON cohort, while one was included in the PPC cohort.

The data revealed that an inpatient stay of more than 3 weeks was significantly more prevalent among WON patients (52%, *p* < 0.001) than among PPC patients (0%). No members of the PPC cohort underwent an inpatient stay of more than 3 weeks. It should be noted that not every patient included in the study underwent an intervention. The vast majority of patients (89%, 17/19) in the PPC cohort underwent an intervention, with the majority (65%, 11/17) undergoing an EUS procedure. In 10 cases, a transgastric drainage was inserted, comprising 5 HotAxios and 5 DPS. Patients with WON were more likely to undergo an ERC for intraductal stenting (59%, 10/17) than transgastric stenting (41%, 7/17).

**Figure 3.**
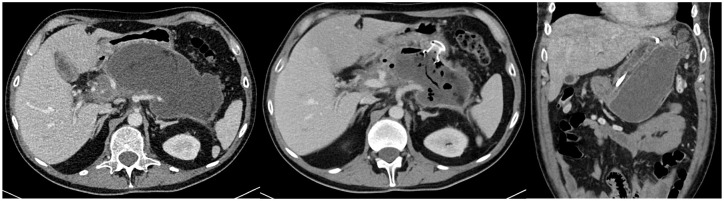
(Left) CT scan with large WON. (Middle) CT scan of the same patient as left after HotAxios implantation. (Right) CT scan of PPC (inlaying nasogastral tube). CT, computed tomography; PPC, pancreatic pseudocyst; WON, walled-off necrosis.

Figure 1 depicts a patient with a sizable WON, representing a local complication of AP, both prior to and following self-expanding metal stent implantation (depicted in the left and middle thirds of Figure 3). Additionally, the figure illustrates a patient presenting with a symptomatic PPC of ethyltoxic aetiology associated with a CP, with an inserted nasogastric tube (depicted in the right third of Figure 3).

Table 3 illustrates the significant findings of the univariate and multivariate regression analyses. No statistically significant results (*p* > 0.05) were obtained for the following variables: sex, BMI, procalcitonin, invasive ventilation, all comorbidities mentioned above, volume of WON or PPC, need for intervention, thrombocytes, need for pain medication, fasting period, tumour and immunosuppressive situation. The WON and PPC cohorts were compared.

**Table 3.** Univariate and multivariate linear regression analyses.

Variables	Univariate	*p* Value	Multivariate	*p* Value
PPC vs WON				
Inpatient stay (>21 days)	0.28 (0.13; 0.57)	**<0.001**	0.85 (0.75; 0.96)	**0.009**
ICU stay	0.11 (0.01; 0.97)	**0.047**		
Chronic pancreatitis	4.07 (1.14; 14.61)	**0.031**		
Ethyltoxic aetiology	6.65 (1.79; 24.73)	**0.005**	5.51 (1.04; 29.25)	**0.045**
Nicotine abuses	3.92 (1.12; 13.76)	**0.032**		
Lipase	0.39 (0.16; 0.96)	**0.041**		
CRP	0.10 (0.01; 0.72)	**0.023**		
Leucocytes	0.11 (0.03; 0.46)	**0.003**		
LDH	0.43 (0.21; 0.88)	**0.021**		
Antibiotic treatment	0.21 (0.05; 0.98)	**0.047**		
Antibiosis ⩽7 days	0.06 (0.01; 0.61)	**0.017**		
Antibiosis monotherapy	0.07 (0.01; 0.39)	**0.003**	0.11 (0.02; 0.76)	**0.025**
Carbapenem	0.03 (0.01; 0.24)	**0.001**	0.04 (0.01; 0.42)	**0.008**
Extrapancreatic localization	0.16 (0.05; 0.50)	**0.001**	0.17 (0.06; 0.53)	**0.002**
Age	0.94 (0.89; 0.99)	**0.029**		

The bold entries indicate a statistical significance (*p* <0.05).

CRP, C-reactive protein; ICU, intensive care unit; LDH, lactate dehydrogenase; PPC, pancreatic pseudocyst; WON, walled-off necrosis.

A direct comparison of the PPC and WON cohorts reveals that the risk of developing a PPC rather than a WON is more than five-fold higher in cases with an ethyltoxic aetiology. Conversely, an inpatient stay of less than 3 weeks is less frequently observed in the PPC cohort. Furthermore, the probability of an extrapancreatic localization of fluid or necrotic collection is also less in PPC than in WON (odds ratio (OR) 0.17).

## Discussion

In this single-centre retrospective study, we analysed the clinical course and risk factors of 46 patients with local complications of severe pancreatitis. The patients had been diagnosed with either acute or CP, and the local complications they suffered included WON and PPC.

Moderately severe to severe AP may manifest with local complications, including PPC, WON, acute peripancreatic fluid collection (APFC) and acute necrotic collection (ANC).^27–29^ The distinction between PPC and APFC lies in the presence of a clearly delineated wall, which is entirely encapsulated in a PPC. By contrast, while no discernible wall encapsulating the collection can be identified in ANC, a WON invariably develops a well-defined inflammatory wall as defined in the Atlanta Classification.^2^ CP can be associated with PPC, CBDS, PDS, pancreatic duct stenosis and other local complications.^30–34^

Approximately 20% of AP patients develop severe AP, which is often associated with a dysfunction or even failure of multiple organs.^35^ Approximately 50% of the total mortality comprises patients who developed an organ failure in the early phase of AP, within the first 7 days from the onset of symptoms.^36^ The mortality of local complications of severe acute or CP ranges around 15% and in case of infection it raises up to 35%.^37–39^ The mortality rate observed in this study cohort is 7%, which is considerably lower than that reported in other studies. One potential explanation for this outcome is the early implementation of endoscopic intervention (either as direct resolution of WON and/or PPC, or as, e.g., symptomatic therapy of cholestasis) and the liberal administration of antibiotics in our tertiary centre. In cases of infected fluid collections or infected wound infections, a common treatment approach involves endoscopic transgastric drainage of the wound or PPC combined with at least two antibiotics for a period exceeding 7 days. Nevertheless, only 74% of patients underwent invasive intervention. The majority of patients who did not undergo invasive intervention presented with a small size of WON or PPC (diameter <5 cm) or no typical symptoms and were additionally characterized by multiple organ failure. In these cases, we elected not to pursue invasive intervention, as the severity of AP or CP was attributable to the multiple organ failure and not directly associated with the local complication. Furthermore, we employed a conventional therapeutic approach comprising iso-osmolar saline solutions, pain management and intensive care via catecholamines, invasive ventilation and other measures in those non-invasive cases. This study shows the distribution of the above-mentioned local complications of both AP and CP.

The correlation with a CP was significantly higher in PPC than in WON (OR 4), which means that our study population had a higher risk of developing a WON in AP than in CP. This finding is also corroborated by other studies, which found that PPC occurs typically in CP, whereas WON can appear in both AP and acute exacerbation of CP, but are more common in AP.^28,40^ The lower morbidity of PPC versus WON can be demonstrated by an OR of 0.3 for a longer inpatient stay (more than 3 weeks).

Moreover, an analysis of a range of laboratory values was undertaken to ascertain any potential correlation with local complications. An elevated level of LDH, leucocytes, CRP and LDH values was found to be associated with a higher proportion of WON compared to PPC (OR 2–16). This indicates that an elevated level of the aforementioned laboratory values (at any point during the hospitalization) is more prevalent in WON than in PPC. These findings are also consistent with those of other studies, where high procalcitonin and CRP levels are effective predictors of infection in acute necrotizing pancreatitis (Tarjan et al.^41^) and high procalcitonin levels predict higher mortality and severity of AP (Mann et al.^42^). An elevated lipase value is controversial as a predictive marker, as it exhibits a higher sensitivity in diagnosing an AP instead of predicting a severe AP.^43,44^

Procalcitonin levels have not been significantly different between our four subcohorts, although it is recognized as a predictor of pancreatitis and is a more reliable indicator of infection in pancreatitis.^41,42,45^

A limitation of this study is its retrospective design and the relatively small number of patients included. Further prospective trials are required to elucidate additional risk factors associated with pancreatitis.

## Conclusion

The findings of this study indicate a relatively low mortality rate (7%) in patients experiencing local complications associated with severe AP and CP. WON may be more frequently associated with AP, carbapenem use, elevated levels of CRP, LDH, lipase and leukocyte count, a higher ICU admission rate and prolonged inpatient stay compared to PPC. PPC may be linked to a reduced incidence of antibiotic treatment, an ethylenic aetiology and an inpatient stay of less than 14 days. It is not necessary to perform invasive treatment procedures on every patient with moderate to severe pancreatitis.

## Declarations

**Ethics approval and consent to participate:** The study was approved by the local Institutional Review Board, the Ethics Committee of the Medical Faculty of the University of Tübingen (IRB number: 279/2020BO2) on 5 May 2020 and was conducted in accordance with the current version of the declaration of Helsinki. Consent to participate was not necessary in the pseudonymized study setting.

**Consent for publication:** Informed consent for publication was not necessary in the pseudonymized study setting.

**Availability of data and materials:** The data supporting the findings of this study are available on request from the corresponding author. The data are not publicly available due to privacy restrictions.

## Abbreviations

ANC acute necrotic collection

AP acute pancreatitis

APFC acute peripancreatic fluid collection

BMI body mass index

CBD common bile duct

CBDS common bile duct stricture

CP chronic pancreatitis

CRP C-reactive protein

CT computer tomography

DEN direct endoscopic necrosectomy

DPS double pigtail stent

ERCP endoscopic retrograde cholangiopan-creatography

EUS endoscopic ultrasound

GP general practitioner

ICU intensive care unit

IQR interquartile range

LAMS lumen apposing metal stent

LDH lactate dehydrogenase

MRI magnetic resonance imaging

OR odds ratio

PDS pancreatic duct lithiasis

PFC pancreatic fluid collection

PPC pancreatic pseudocyst

SD standard deviation

SEMS self-expanding metal stent

WON walled-off necrosis
